# Lighting up Neuroanatomy

**DOI:** 10.3389/fnins.2016.00293

**Published:** 2016-06-23

**Authors:** Kathleen S. Rockland

**Affiliations:** Department of Anatomy & Neurobiology, Boston University School MedicineBoston, MA, USA

**Keywords:** quantitative neuroanatomy, Purkinje cells, cerebellum, light sheet microscopy, image analysis

“Contemporary neuroscience is in urgent need of a new generation of neuroanatomical techniques allowing scalable, reliable, specific, and quantitative analysis of macroscopic portions of brain tissue with cellular or sub-cellular resolution.” In this first sentence of their Discussion, Silvestri et al. (2015) succinctly set forth the key issues of their contributing article; namely, visualization of Purkinje cells in 3-D volumes, and the methods for doing so.

The importance of rigorous quantitative analysis in neuroscience has long been recognized as an essential baseline against which to compare and interpret species differences, developmental changes, and pathological changes, among other processes. Counting, however, is not easy, and incorrect sampling methods have more than once produced fundamentally incorrect results—notably, the now discredited belief that there is no neuronal loss in normal aging (reviewed in Guillery, 2002), or that there are “ten times” more glia than neurons (reviewed in Hilgetag and Barbas, 2009). A particular challenge in neuroanatomy has been the problem of compiling accurate quantitative data about inherently 3-dimensional structures from essentially 2-dimensional histological sections. The standard way of dealing with this problem has been the application of specific stereological procedures. Even when combined with some degree of automation, however, stereology is notoriously labor intensive, and provides only limited information about spatial localization and pattern.

A rapidly emerging alternative approach in quantitative neuroanatomy is direct 3-dimensional visualization. In itself, this is not new; for example, whole mount preparations reacted for various antigens have demonstrated an intricate compartmentalization of the cerebellar cortex (White et al., 2012; Cerminara et al., 2015); and reconstructed stacks of consecutive histology slides have successfully demonstrated the complex striosomal architecture (Mikula et al., 2009). The newer techniques, although not without their own problems, offer faster processing, better standardization, and the potential for integration across cellular and subcellular spatial scales.

Several volumetric approaches are available. Serial two-photon tomography is a block-face approach developed in the early 2000's and now in routine use for whole mouse brains (Ragan et al., 2012; Kim et al., 2015). The main features (reviewed in Amato et al., 2016) include straightforward specimen preparation, minimal tissue processing, automated and robust data acquisition, and high resolution 3-D reconstructions.

In confocal light sheet microscopy (LSM), the approach used by Silvestri et al., the sample is illuminated with a thin sheet of light and optically sectioned, typically in a wide-field detection scheme. Millions of pixels are collected simultaneously by a camera instead of sequentially as in standard confocal or two-photon microscopy. Several modifications have been developed, each with some tradeoffs between the physical width of the lightsheet and the size of the specimen that can be imaged. Thin lightsheets (5 μm − <1 μm) provide the best axial resolution, but are limited to small samples. Physical specifications and configuration are reviewed in Silvestri et al., as well as in a number of other sources, reflecting the intense interest in this burgeoning field (Santi, 2011; Reynaud et al., 2015; Richardson and Lichtman, 2015; Tomer et al., 2015).

LSM entails a number of interrelated steps, which are clearly detailed in Silvestri et al. As shown in their figure 1, the experimental pipeline for large-volume quantitative neuroanatomy consists of tissue clearing (for which there are a number of options; see also Richardson and Lichtman, 2015), actual imaging, image stitching, automatic cell localization, and further statistical analysis. None of these steps are routine and all are best viewed as still under development. The Authors are careful to emphasize that what they call large-scale quantitative neuroanatomy is still in its infancy (last paragraph of the Discussion) and requires both aggressive quality control and interactive testing of visualization and annotation tools.

A major factor in the new pipeline is (for neuroscientists) the unaccustomedly huge size of the datasets, requiring sophisticated analysis capabilities and a disposition for multi-disciplinary interactions (see Reynaud et al., 2015: “The technology is ready to assist biologists in tackling scientific problems, but are biologists ready for it?”). On the positive side, these exigencies are encouraging the fast emergence of a new mindset, eager to accept the interdisciplinary challenges.

Silvestri et al. take as a specific application the number and arrangement of Purkinje cells in the mouse cerebellum. This is an attractive target for multiple reasons: Purkinje cells are large, unambiguously identifiable by virtue of their size and location, they are moderately well-isolated, and variations have been reported in instances of human pathological conditions. The total number of Purkinje cells calculated in the selected P10 L7-GFP mouse is given as 221,107. This accords well with the estimates derived from unbiased stereology (e.g., Woodruff-Pak, 2006). Silvestri et al., report further geometric data, acquired by a clustering algorithm. Setting the number of nearest neighbors equal to 3, the Authors report that 94% of identified Purkinje cells occur in large clusters, that 1131 clusters are made up of fewer than 100 cells, and that there are 1389 isolated neurons.

This analysis is reminiscent of similar spatial analysis of apical dendritic bundles in cerebral cortex. In several studies, tangential sections through cortical layer 3 have revealed an organized array of dendritic clusters, containing about 50 dendrites in humans or 30 in macaque monkeys (Peters, 1994; Gabbott, 2003). This and other quantitative/pattern questions are well-suited to re-investigation with the new, faster protocols.

In summary, large-scale quantitative neuroanatomy, via 3-D data acquisition and analysis, has emerged as an important new tool. More than tool, it is spearheading a welcome “user's group”/new community with shared interests and priorities. The energy is palpable, and bodes well for renewed attention to basic neuroanatomical issues. It effectively rebuts, via standardization and quantification, the old accusation of neuroanatomy as “descriptive.”

Major challenges undoubtedly lie ahead. Technical: can the current tradeoff of resolution and field width in LSM be narrowed? What are the effective ways for dealing with very large datasets? Sociological: how do we optimally combine the traditional small, PI driven lab with large centers or core facilities? Databases with ideas? Conceptual: What issues are the most tractable to this approach? How do we achieve more effective methodological integration; for example, gene expression data or receptor distribution along with cytoarchitecture? How do we incorporate cell type and connectivity data? How do we incorporate the temporal domain? Questions notwithstanding, this is an impressive new direction, and continued progress on all fronts can be expected.

## Author contributions

All aspects of this Commentary article have been solely carried out by KR.

### Conflict of interest statement

The author declares that the research was conducted in the absence of any commercial or financial relationships that could be construed as a potential conflict of interest.
